# Professional Training and Personal Development: Lessons From 10 Years of Intergenerational Learning

**DOI:** 10.1093/geroni/igaa057.003

**Published:** 2020-12-16

**Authors:** David Steitz

**Affiliations:** Nazareth College, Rochester, New York, United States

## Abstract

This presentation will highlight a ten-year collaboration between the Nazareth College Gerontology Program and St. John’s Senior Services – the St. John’s Collaborative for Intergenerational Learning (SCIL). Specifically, semester-long intergenerational coursework (Adulthood & Late Life, Issues in Aging, Aging & Community Service) and community-based service projects will be showcased with a focus on design, implementation, assessment, and impact. Emphasis will be placed on the reciprocity of these collaborations, the personal and professional benefits of these exchanges for our students, and the subsequent impact on the community partners and the individuals they serve. Various models of intergenerational learning as well as our new intergenerational residency program will also be discussed.

